# Biodegradation of Low Density Polyethylene by the Fungus *Cladosporium* sp. Recovered from a Landfill Site

**DOI:** 10.3390/jof9060605

**Published:** 2023-05-24

**Authors:** Zhu Gong, Long Jin, Xingye Yu, Baoteng Wang, Shuang Hu, Honghua Ruan, Yun-Ju Sung, Hyung-Gwan Lee, Fengjie Jin

**Affiliations:** 1 Co-Innovation Center for Sustainable Forestry in Southern China, College of Biology and the Environment, Nanjing Forestry University, 159 Longpan Road, Nanjing 210037, China; gongzhu01@njfu.edu.cn (Z.G.); isacckim@alumni.kaist.ac.kr (L.J.);; 2 Technology Transfer Centre, Korea Research Institute of Bioscience & Biotechnology (KRIBB), Daejeon 34141, Republic of Korea; 3 Cell Factory Research Centre, Korea Research Institute of Bioscience & Biotechnology (KRIBB), Daejeon 34141, Republic of Korea

**Keywords:** biodegradation, *Cladosporium* sp. CPEF-6, LDPE degradation, laccase, plastic-degrading fungi

## Abstract

Low density polyethylene (LDPE) has been widely used commercially for decades; however, as a non-degradable material, its continuous accumulation has contributed to serious environmental issues. A fungal strain, *Cladosporium* sp. CPEF-6 exhibiting a significant growth advantage on MSM-LDPE (minimal salt medium), was isolated and selected for biodegradation analysis. LDPE biodegradation was analyzed by weight loss percent, change in pH during fungal growth, environmental scanning electron microscopy (ESEM), and Fourier transformed infrared spectroscopy (FTIR). Inoculation with the strain *Cladosporium* sp. CPEF-6 resulted in a 0.30 ± 0.06% decrease in the weight of untreated LDPE (U-LDPE). After heat treatment (T-LDPE), the weight loss of LDPE increased significantly and reached 0.43 ± 0.01% after 30 days of culture. The pH of the medium was measured during LDPE degradation to assess the environmental changes caused by enzymes and organic acids secreted by the fungus. The fungal degradation of LDPE sheets was characterized by ESEM analysis of topographical alterations, such as cracks, pits, voids, and roughness. FTIR analysis of U-LDPE and T-LDPE revealed the appearance of novel functional groups associated with hydrocarbon biodegradation as well as changes in the polymer carbon chain, confirming the depolymerization of LDPE. This is the first report demonstrating the capacity of *Cladosporium* sp. to degrade LDPE, with the expectation that this finding can be used to ameliorate the negative impact of plastics on the environment.

## 1. Introduction

Plastics are extensively employed in agriculture, manufacturing, and daily life because of their low cost, high strength, and excellent durability [1]. Since the 1950s, global production of plastics has grown extremely fast [2], and global consumption of plastics is growing at an annual rate of 12%, with approximately 150 million tons of composite polymers produced worldwide each year [3]. In recent years, it has emerged as one of the fastest-growing industries in the global industrial sector. From 1950 to 2015, an estimated 630 million metric tons of plastic waste have been generated, 79% of which has been deposited in landfills or spilled into the natural environment [4]. Because of its non-biodegradable nature, plastic can last a long time without losing its properties. Under normal conditions, it takes more than 100 years for the polymer to mineralize [5]. In some instances, they can produce toxic compounds that are detrimental to animals and cause physical damage [6]. Significantly, once plastics are released into the environment, they degrade into smaller fragments through weathering and other decomposition processes [7], which can adsorb organic pollutants and act as carriers for many toxic chemicals, providing a way to contaminate the soil environment [8].

Low density polyethylene (LDPE) accounts for 60% of the total production of plastic bags, which is the most common solid waste [9]. LDPE is a semicrystalline solid with several properties, such as chemical resistance, durability, rigidity, and flexibility, even at low temperatures [10]. Due to these versatile properties, LDPE is widely used in almost any industrial, agricultural, or domestic market [11]. At present, plastic waste disposal methods include landfilling, incineration, and recycling. However, these methods are not only considered expensive, but also have harmful effects on human life [12]. Of these, bioremediation degrades toxic pollutants into non-toxic metabolites via microorganisms such as bacteria and fungi, with the end products usually being carbon dioxide and water [6]. Bioremediation is an environmentally friendly and cost-effective technique that is the best approach for eliminating many environmental pollutants [13].

Until now, a wide range of microorganisms, including bacteria and fungi, have been investigated for plastic biodegradation. Compared to bacteria, fungi grow faster in soil and can expand and penetrate to other locations through the growth of mycelial distribution, making them more suitable for LDPE degradation [14]. As fungi search for nutrients, they extend in the substrates through filamentous network structures, thus exploring and growing in places that are more difficult for other microorganisms to reach [14]. Fungi have developed an exceptional ability to adapt to changing environments and to break down and utilize a variety of contaminants [15]. Consequently, fungi play a vital role in the degradation and mineralization of various environmental pollutants via various metabolic pathways and chemical reactions [16].

Fungal metabolic diversity and its ability to degrade complicated chemicals suggest that fungi have the potential to degrade plastics [15]. To date, a number of plastic-degrading fungi have indeed been identified, including *Trichoderma viride*, *Aspergillus niger*, *Aspergillus terreus*, *Paecilomyces varioti*, *Penicillium ochrochloron*, and *Aureobasidium pullulans*, which are all reported to have LDPE-degrading abilities [17,18]. The fungus *Neopestalotiopsis phangngaensis* showed significant biodegradation capacity after 90 days of incubation in LDPE flakes alone as the sole carbon source [19]. Some studies have shown that the endophytic fungi *Paecilomyces lilacinus*, *Aspergillus* sp., and *Lasiodiplodia theobromae* isolated from plants were co-cultured with gamma-treated LDPE films. After 90 days of co-culture, these fungi generated laccase while utilizing LDPE. The reduction in the sheets’ intrinsic viscosity and average molecular weight indicated these fungi’s ability to degrade LDPE [20]. In another study, after 105 days of incubation with LDPE, both *Penicillium* sp. and *A. terreus* screened from mangrove soil showed degradation potential. In particular, the weight of the LDPE bag samples treated with *A. terreus* was reduced by 24%, which proved its superiority. In this process, these two fungi also produced laccase and peroxidase [21]. These results suggest that fungi have great potential for LDPE degradation, making it critical to find more LDPE-degrading fungi.

Fungi produce a wide range of non-specific enzymes that catalyze the degradation of environmental pollutants [22]. These enzymes improve the hydrophilicity of polyethylene (PE), which allows microorganisms to attach to the PE surface [23]. In recent years, laccases, manganese peroxidase (MnP), and lignin peroxidase, among others, have been reported to be involved in the biodegradation of PE [24]. Laccases are a class of enzymes belonging to the family of blue copper oxidases and are multicopper monomeric glycoproteins [25]. For example, laccases and MnP secreted by *Bacillus cereus* were reported to degrade UV-irradiated PE [26]. In addition to bacteria that secrete laccase for PE degradation, *Aspergillus fumigatus* B2.2, screened by Muhonja et al., was also found to secrete laccase for PE degradation [27]. Laccase exhibits a broad range of substrate specificities. Therefore, testing whether a strain produces laccase could be a way to identify whether a strain is PE degradable or not.

The fungal genus *Cladosporium* includes a large number of species [28] belonging to the family *Cladosporiaceae*, which feature distinctive coronate structures in their conidiogenous loci and conidial hila, which have a central convex dome surrounded by a raised periclinal rim [29]. *Cladosporium* spp. are widely distributed in nature and are usually isolated from food, soil, air, plants, and textiles, as endophytes, plant pathogens, or hyperparasites on other fungi [28,30]. Recent studies have demonstrated that polyurethane (PU), as the sole carbon source, can be degraded by the fungus *Cladosporium* sp. [31]. PU is a synthetic polymer that is widely used throughout the world [32]. Studies have already identified a highly active PU-degrading fungal strain, *Cladosporium* sp. P7, and suggested a possible metabolic mode of PU degradation by P7 through ester or polyurethane bonds [31]. In another study, researchers isolated a fungal strain from the deep sea. The fungal species *Cladosporium halotolerans* can grow in mineral media with polyester PU as the only carbon source [33]. These findings imply that the fungi *Cladosporium* spp. have the potential to degrade complex polymers. However, so far, no relevant investigations have yet demonstrated that *Cladosporium* spp. have the ability to degrade LDPE. Physical and chemical pretreatments can significantly enhance the bioavailability of the polymer [34]. Thermal degradation is basically a “molecular deterioration due to overheating”, where high temperatures trigger the splitting of chemical bonds. The long chain backbones of polymers initiate breaking of the bond, and these components interact to change the properties of the polymers [35]. The majority of polymers in the environment are exposed to weathering conditions that result in mechanical, thermal, and/or chemical transformations that affect the biodegradation of these polymers [34]. Therefore, pretreatment can be employed as a necessary prerequisite to promote the effective biodegradation of plastic fragments.

This investigation aims to isolate and characterize LDPE-degrading fungi recovered from soil. As a result, *Cladosporium* sp. CPEF-6, a fungus capable of degrading LDPE, was isolated. CPEF-6 has the ability to degrade LDPE and can use LDPE as the sole carbon source. The biodegradation degree of LDPE sheets was determined by monitoring the changes in various physicochemical parameters such as weight, pH, surface morphology, and chemical composition of the LDPE film sheets after incubation with CPEF-6 for 30 days under laboratory conditions. In order to improve the degradation efficiency, additional heat treatment of LDPE sheets was carried out. Finally, the production of laccase by CPEF-6 was investigated. This study provides information on the microbial degradation of LDPE and expands the library of fungal degradation of LDPE.

## 2. Materials and Methods

### 2.1. Soil Landfill Experiments

Soil samples from different sources were tumbled to mix, and then commercially available plastic wrap fragments and plastic sealed bag fragments were added to this soil sample for enrichment, simulating the natural environment for sprinkling and flipping. After 100 days, plastic fragments with more adherent soil were selected for follow-up experiments. 

### 2.2. LDPE Sample Preparation for Biodegradation Assay

LDPE microplastic granules (Shanghai Macklin Biochemical, Shanghai, China) were employed with a melting index of 20–30 g/10 min and a particle size of ~1000 mesh. The LDPE microplastic granules were weighed, put into Petri dishes, and exposed to UV irradiation for 12 h in the ultra-clean bench. LDPE Clear Open End Bags (Aladdln Industrial Corporation, Shanghai, China) with a thickness of 0.038 mm were cut into 3 cm × 3 cm pieces, weighed, recorded, and placed separately in sterile Petri dishes for further use. The untreated sheets (U-LDPE) were soaked and sterilized with 75% ethanol for 3 h in the ultra-clean bench and rinsed several times with sterile water. Subsequently, the sheets were air dried in the ultra-clean bench and sterilized with UV for 12 h. Thermal treatment sheets (T-LDPE) were prepared as follows: The sheets were heat-treated to induce a change in LDPE structure. The sheets were then heated at 75 °C for 7 days in a hot air oven under dry and dark conditions. The sterilization method was the same as U-LDPE.

### 2.3. Media and Culture Conditions

The minimal salt medium (MSM) was composed of the following (in g/L): KH_2_PO_4_ (1.0 g), Na_2_HPO_4_ (1.5 g), NH_4_Cl (2.0 g), CaCl_2_·2H_2_O (0.1 g), KCl (0.15 g), MgSO_4_·7H_2_O (0.2 g), FeSO_4_·7H_2_O (0.01 g), ZnSO_4_·7H_2_O (0.01 g), and MnSO_4_·H_2_O (0.001 g), in 1 L of distilled water. The micro carbon source medium (MCM) was made up of the following ingredients (in g/L): yeast extract (0.5 g), (NH_4_)_2_SO_4_ (2.0 g), FeSO_4_·7H_2_O (1.0 g), MgSO_4_·7H_2_O (1.0 g), CuSO_4_·5H_2_O (0.1 g), MnSO_4_·H_2_O (0.1 g), and ZnSO_4_·7H_2_O (0.1 g), in 1 L of distilled water. Their agar plates were prepared by adding 15 g of agar to the 1 L liquid medium above. Fungal cultures of the LDPE degrading strains were maintained on potato dextrose agar medium (PDA; BD Difco, Sparks, MD, USA). Potato dextrose agar medium (PDA; BD Difco, Sparks, MD, USA) was used to maintain the growth of the LDPE degrading fungus. Liquid medium culture experiments were carried out in 250 mL flasks, all of which were cultured at 30 °C with the rotation speed of 150 rpm.

### 2.4. Isolation and Screening of the LDPE Degrading Fungi

Soil solution with three different dilution concentrations (10^−4^, 10^−5^, and 10^−6^) was inoculated into 100 mL of the MCM medium for acclimatization, in which 1g sterile LDPE microplastic granules were added as a carbon source. After 30 days of incubation, LDPE microplastic granules were taken out and put into 100 mL MSM medium for secondary enrichment. After 30 days of incubation, 500 µL of enrichment solution was transferred and dispersed on the MSM plate with LDPE sheets as the sole carbon source. Bacterial growth was inhibited by 1% streptomycin added to the MSM plate. Then, single colonies were selected and repeatedly transferred to fresh PDA plates until pure isolates could be obtained. The single colonies were stored in glycerol at −80 °C. To further screen the fungi with strong LDPE degrading ability, the isolates were cultivated in MSM-LDPE (U-LDPE sheets) medium for 30 days. Their biodegradability of LDPE was preliminarily determined by observing the growth of microorganisms and the attachment of the strain on LDPE sheets. 

### 2.5. Molecular Identification of LDPE Degrading Fungus

Genomic DNA was directly extracted from the mycelium of the fungal isolates using a genomic DNA preparation kit (Toyobo, Osaka, Japan). The fungal isolates were identified based on internal transcribed spacer (ITS) region. PCR amplification was carried out using the universal primers ITS4: 5′-TCCTCCGCTTATTGATATGC-3′ and ITS5: 5′-GGAAGTAAAAGTCGTAACAAG-3′ for the ITS region [33]. DNA sequences obtained from sequencing were searched for similar sequences in the NCBI database, and multiple sequence alignment was performed according to the principle of maximum homology. The sequences of the obtained ITS genes were compared using Clustal_X v.2.1 [36], and gene editing was performed using BioEdit. A phylogenetic tree was constructed using the neighbor-joining method [37] with bootstrap values determined by 1000 replicates in MEGA7 [38].

### 2.6. Biodegradation of the LDPE Sheets

The biodegradation ability of the selected strains was further evaluated using LDPE sheets. The spore suspension (500 µL) and the sterilized LDPE sheets were placed in 100 mL MSM liquid medium. They were then incubated in a shaker at 30 °C for 30 days. Samples without the addition of spore suspension were used as the controls. Each experiment was performed in triplicates.

#### 2.6.1. Weight Loss Measurement

The most common method for determining the rate of biodegradation is weight loss. To assess weight change, LDPE sheets were collected after 30 days of fungal treatment and washed overnight with 2% (*v*/*v*) sodium dodecyl sulfate (SDS) to remove biofilms. Then, the LDPE sheets were cleaned with 75% ethanol, rinsed 3 to 4 times with distilled water, and dried in an oven. Finally, the dried LDPE sheets were weighed and the weight loss percentage (%) was calculated using the formula below.

(1) $$\mathrm{Weight}\;\mathrm{loss}\;\mathrm{percentage}=\frac{\left(\mathrm{initial}\;\mathrm{weight}\right)-\left(\mathrm{final}\;\mathrm{weight}\right)}{\;\mathrm{initial}\;\mathrm{weight}}\;\times \;100\%$$

#### 2.6.2. Change in pH

The pH changes of the culture solution represent the metabolic activity of the fungal strains because the polymers degrade in the process of fungal metabolism [39]. Throughout incubation, changes in pH of individual fungal medium were recorded periodically at 7-day intervals.

#### 2.6.3. Surface Morphology of LDPE Films

The surface morphology of untreated and heat-treated LDPE (U-LDPE and T-LDPE, respectively) sheets were analyzed using environmental scanning electron microscopy (FEI Quanta 200) (FEI Company, Hillsboro, OR, USA). Dehydrated samples were dried, mounted, and then sputter-coated with gold for ESEM analysis.

#### 2.6.4. Fourier Transformed Infrared (FTIR) Spectroscopic Analysis

The chemical structure of the U-LDPE and T-LDPE sheets was studied comparatively using Fourier transformed infrared spectroscopy (Bruker VERTEX 80 V) (Bruker Corporation, Bremen, Germany).

### 2.7. Laccase Activity

2-2′-azinobis-(3-ethylbenzothiazoline-6-sulfonic acid) (ABTS) was used to measure the activity of laccases secreted by the isolate CPEF-6 during LDPE degradation [40]. Subsequently, 1.0 mL of 0.5 mM ABTS solution was mixed with 1 mL of 10 mM sodium acetate buffer (pH 4.6) and incubated at 37 °C. After this, 1 mL of the mixed culture supernatant was added to start the reaction. After 10 min, the absorbance was recorded directly at 420 nm using a Nanodrop 2000C spectrophotometer. The micromoles of oxidized ABTS were calculated using an Σ420 value of 36,000 (mol oxidized ABTS)^−1^ cm^−1^. The unit (U) of enzyme activity is defined as the quantity of the enzyme required to alter absorbance (420 nm) by 0.001 per minute at 37 °C. The formula is as follows:

(2) $$U(U/L)=\frac{\Delta \mathrm{OD}420\;\times \;10^{6}\;\times \;n}{V\;\times \;\Delta t}$$

V was the volume of culture liquid (mL) and n was the dilution factor.

### 2.8. Statistical Analysis

All the experiments were carried out in triplicates. Statistical analysis was performed using SPSS software, which was used to conduct one-way ANOVA. The data obtained were presented as mean ± standard deviation (SD). A *p* value < 0.05 was considered as a statistically significant difference.

## 3. Results

### 3.1. Isolation and Screening of LDPE Degrading Fungus

**Figure 1:**
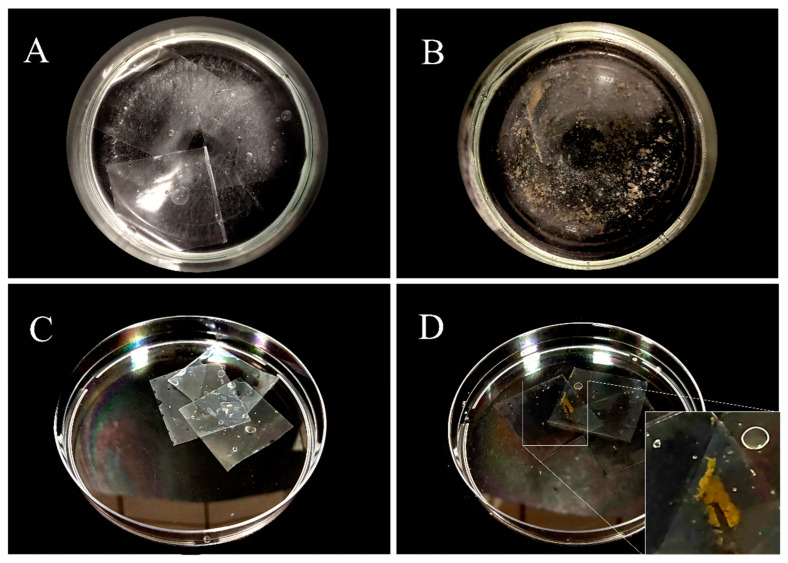
Growth of strain CPEF-6 in MSM-LDPE medium and attachment to LDPE sheets. (**A**) The turbidity of the medium without spore suspension and (**B**) spore suspension with CPEF-6 added. (**C**) LDPE sheets treated without spore suspension were removed after 30 days of incubation. (**D**) LDPE sheets with CPEF-6 added to the spore suspension removed after 30 days of incubation; the white box indicates colonies attached to the LDPE sheets.

A total of 54 fungal strains were isolated from MSM-LDPE plates after secondary enrichment using LDPE as the sole carbon source. The predominant isolates were *Aspergillus* sp., *Penicillium* sp., *Talaromyces* sp., *Alternaria* sp., and *Arthrinium* sp. The first stage of PE biodegradation is the adhesion of microorganisms to their surface. To screen for fungi with the ability to biodegrade LDPE, we described the amount of fungal growth and the attachment of strains on the LDPE sheet (Table S1). The results showed that the culture medium of the blank control was clearer (Figure 1A) and there was no attached mycelium (Figure 1C) on the LDPE sheets, whereas CPEF-6 had green mycelial growth in MSM-LDPE medium (Figure 1B) and there was obvious green mycelium attached to the LDPE sheets co-cultured with CPEF-6 (Figure 1D). Among these fungi, the growth of strain CPEF-6 and its attachment to the LDPE sheets were more obvious. Therefore, the fungal strain CPEF-6 was selected as a potential LDPE degrading fungus since it grew better than all other strains in MSM-LDPE liquid media.

### 3.2. Morphological and Phylogenetic Identification of LDPE Degrading Fungus CPEF-6

**Figure 2:**
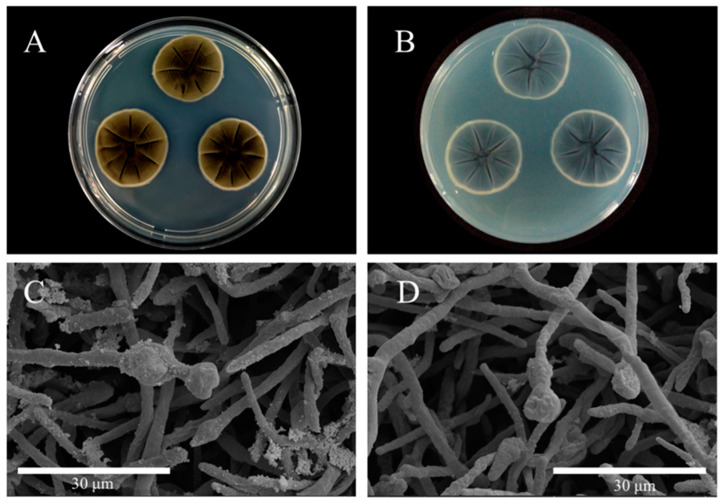
Morphological features of strain CPEF-6. (**A**) The colony morphology of strain CPEF-6 after 7 days of incubation on the obverse PDA plate. (**B**) The colony morphology of strain CPEF-6 after 7 days of incubation on the reverse PDA plate. (**C**,**D**) Conidiophore of strain CPEF-6 (from **A** and **B**, respectively).

**Figure 3:**
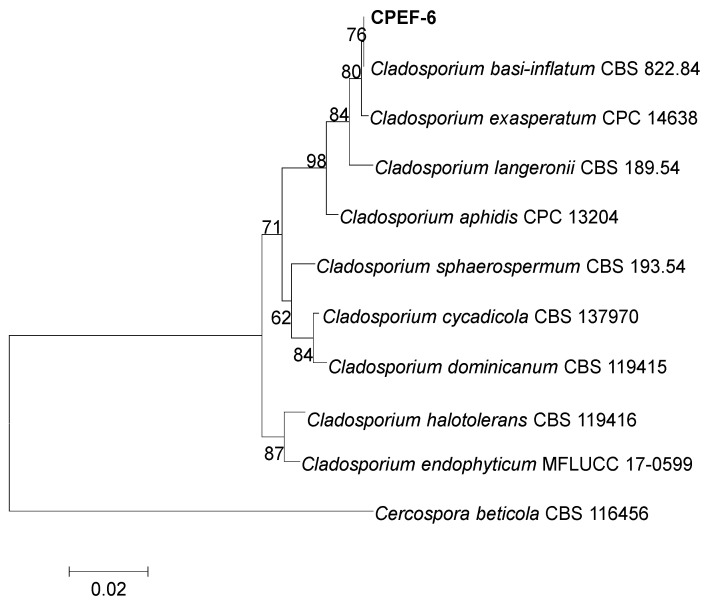
Neighbor-joining tree of the fungal genus *Cladosporium* based on ITS sequences. The tree was rooted to *Cercospora beticola* (CBS 116456). The evolutionary history was inferred using the neighbor-joining method [37]. The phylogenetic tree was constructed using the MEGA 7 program. The supported values from the 1000 bootstrap copies are illustrated under their respective branches.

The isolate CPEF-6 grown on PDA reached 25–27 mm in diameter after 7 days at 30 °C (Figure 2A,B). Colonies on PDA are greenish–olivaceous to grey–olivaceous due to profuse sporulation and mycelium, powdery to fluffy or felty, reverse iron-grey to dark leaden-grey, margin narrow, white, feathery, aerial mycelium diffuse, loose, fluffy, without prominent exudates, sporulation profuse, mainly in the colony centre (Figure 2). The fungal isolates were identified based on the sequence analysis of ITS region homology. ITS gene sequences were retrieved from GenBank (Table S2), including closely related *Cladosporium* sp. and the sequence of strain CPEF-6 (accession no. OQ651281). Finally, strain CPEF-6 was identified as *Cladosporium basi-inflatum* by using the neighbor-joining method (Figure 3).

Furthermore, another fungal isolate, designated CPEF-7 recovered in this study, grew rapidly and produced a considerable number of conidia, and the colonies of CPEF-7 on PDA were 72–76 mm in diameter after 5 d, 30 °C (Figure S1A,B). Colonies on PDA are greyish turquoise or dark turquoise to dark green or dull green (Figure S1A). The reverse color was creamy and yellow to orange (Figure S1B). Conidiation was abundant and rarely less abundant (Figure S1C,D). Colony texture was velutinous (Figure S1A) [41]. The fungal isolate was identified using the sequence analysis of ITS region homology. ITS gene sequences were retrieved from GenBank (Table S3), including *Aspergillus fumigatus* and the sequence of our isolate strain CPEF-7 (accession no. OQ651969). Finally, CPEF-7 was identified as *A. fumigatus* based on the neighbor-joining method (Figure S2). In this study, the strain CPEF-7 was used as a reference to determine and evaluate the degradability of CPEF-6 since *A. fumigatus* has been proven to have strong LDPE degradation ability in other studies. 

### 3.3. Weight Loss of LDPE Sheets

**Figure 4:**
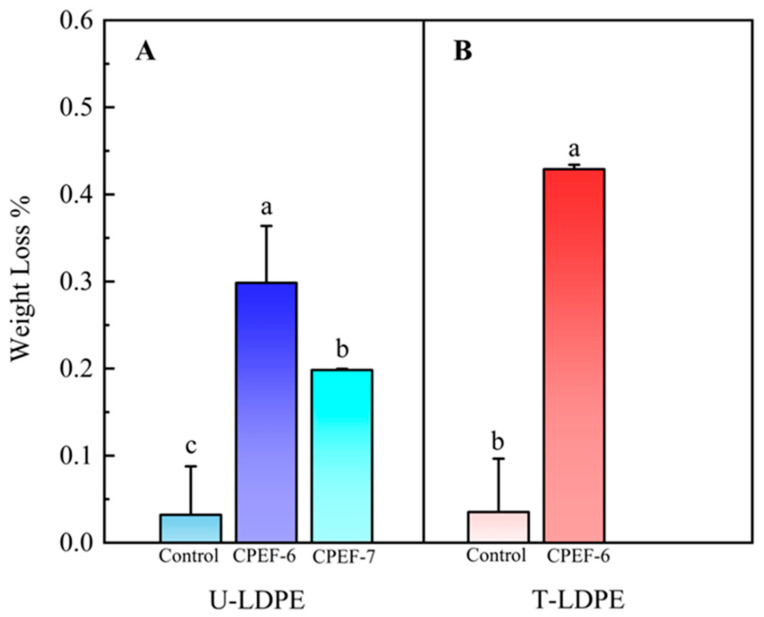
(**A**) The percentage weight loss of U-LDPE sheets by CPEF-6 and CPEF-7 after 30 days of biodegradation assay. (**B**) The percentage weight loss of T-LDPE by CPEF-6 after 30 days of the biodegradation assay. Error bars represent the standard error (*n* = 3). Different letters on the error bars represent significant change within sampling days (*p* < 0.05).

In order to evaluate the ability of fungi to degrade LDPE, the dry weight of the residual polymer was measured to compute the weight loss of LDPE during the biodegradation process. The decrease in LDPE sheets weight was attributed to the fungus using LDPE as its sole carbon source during growth. In this experiment, 3 cm × 3 cm LDPE sheets were used for degradation studies and *A. fumigatus* CPEF-7 was used as the reference strain to compare and evaluate the degradation ability of CPEF-6. After 30 days of treatment, both fungal isolates CPEF-6 and CPEF-7 showed a reduction in the weight of the LDPE film by 0.30 ± 0.06% and 0.20 ± 0.01%, respectively, as compared to the blank control (Figure 4A). This result validated the potential of CPEF-6 in the degradation of LDPE, and its degradation capacity exceeded that of *A. fumigatus* CPEF-7.

In order to change the structure of LDPE and make it more degradable, the LDPE sheets were subjected to heat treatment (T-LDPE). The results showed that after 30 days of co-culture with CPEF-6, the weight of T-LDPE sheets decreased by 0.43 ± 0.01% (Figure 4B), which was higher than that of untreated (U-LDPE) sheets. 

### 3.4. Changes in pH during LDPE Degradation

**Figure 5:**
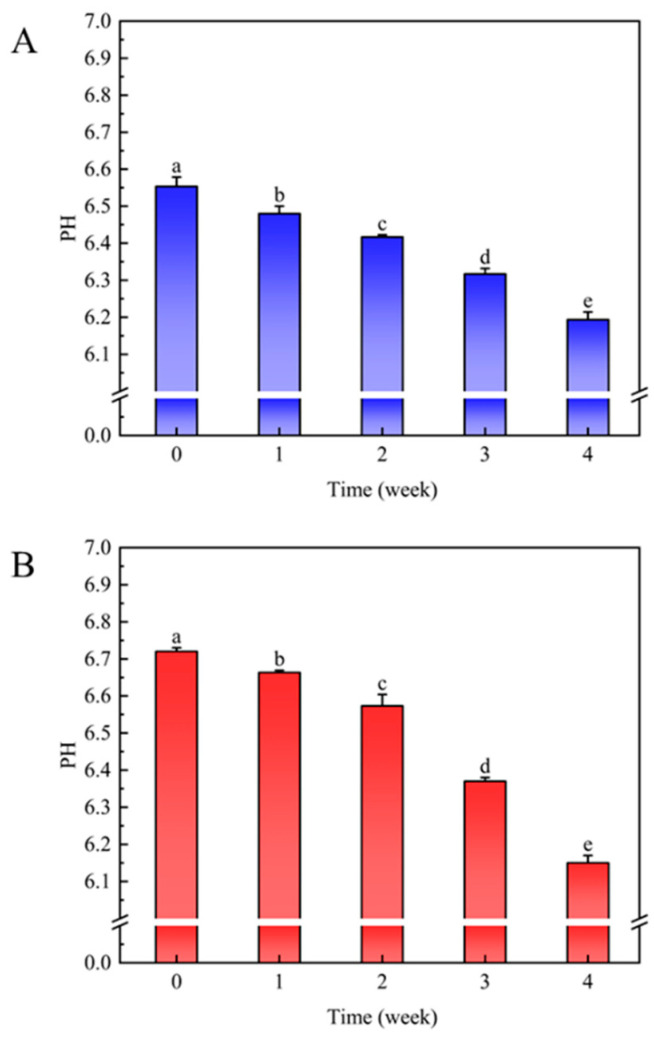
The change in pH during the degradation of U-LDPE and T-LDPE by CPEF-6. (**A**) The change in pH during the degradation of U-LDPE by CPEF-6. (**B**) The change in pH during the degradation of T-LDPE by CPEF-6. Error bars represent the standard error (*n* = 3). Different letters on the error bars represent significant change within sampling weeks (*p* < 0.05).

The change in pH during the degradation of U-LDPE and T-LDPE by CPEF-6 are shown in Figure 5. pH values were measured every 7 days during the degradation test. The results showed that the pH of the CPEF-6 culture medium gradually declined throughout the 30 days of LDPE degradation from neutral to weakly acidic. The initial pH of the U-LDPE biodegradation experiment was 6.55 ± 0.03, and after 30 days of incubation with CPEF-6, the pH changed to 6.19 ± 0.02 (Figure 5A). In the same way, the initial pH of T-LDPE incubated with CPEF-6 was 6.72 ± 0.01, which dropped to 6.15 ± 0.02 after 30 days (Figure 5B). According to these findings, the pH change during T-LDPE degradation is more significant than that of U-LDPE degradation.

### 3.5. Environmental Scanning Electron Microscopy Observation of LDPE Biodegradation

**Figure 6:**
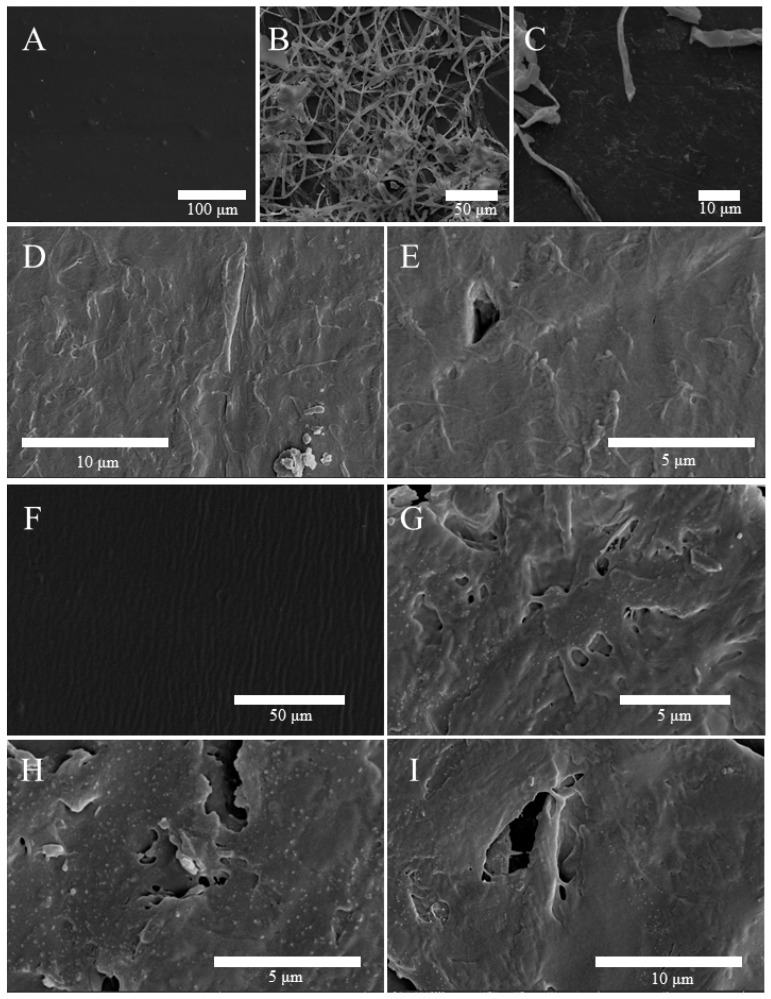
ESEM micrographs of U-LDPE and T-LDPE sheets. (**A**) The smooth U-LDPE polymer surface as control (without fungal treatment). (**B**–**E**) The rough U-LDPE surface of the sheets due to the fungal treatment with CPEF-6. (**F**) The smooth T-LDPE polymer surface as control (without fungal treatment). (**G**–**I**) The rough T-LDPE surface of the sheets due to the fungal treatment with CPEF-6.

The change in morphology of the LDPE surface after 30 days of incubation with the fungus was observed by environmental scanning electron microscopy. The surfaces of the U-LDPE sheets inoculated with the CPEF-6 became rough and uneven, with visible surface corrosion and erosion holes, clear cracks, and particulate matter formation (Figure 6D,E). Meanwhile, mycelium adherence to the LDPE sheets could also be observed (Figure 6B,C). In contrast, the surface of the LDPE sheets in the un-inoculated treatments remained smooth and intact without any changes (Figure 6A). In addition, we also found that after inoculation with CPEF-6, the surface corrosion of the heat-treated T-LDPE sheets was more serious, with more obvious cracks and larger pores (Figure 6G–I).

These findings suggest that CPEF-6 has the ability to degrade polyethylene, and thus can cause certain damage to the surface of LDPE, while heat-treated LDPE is more susceptible to degradation.

### 3.6. FTIR Analysis

**Figure 7:**
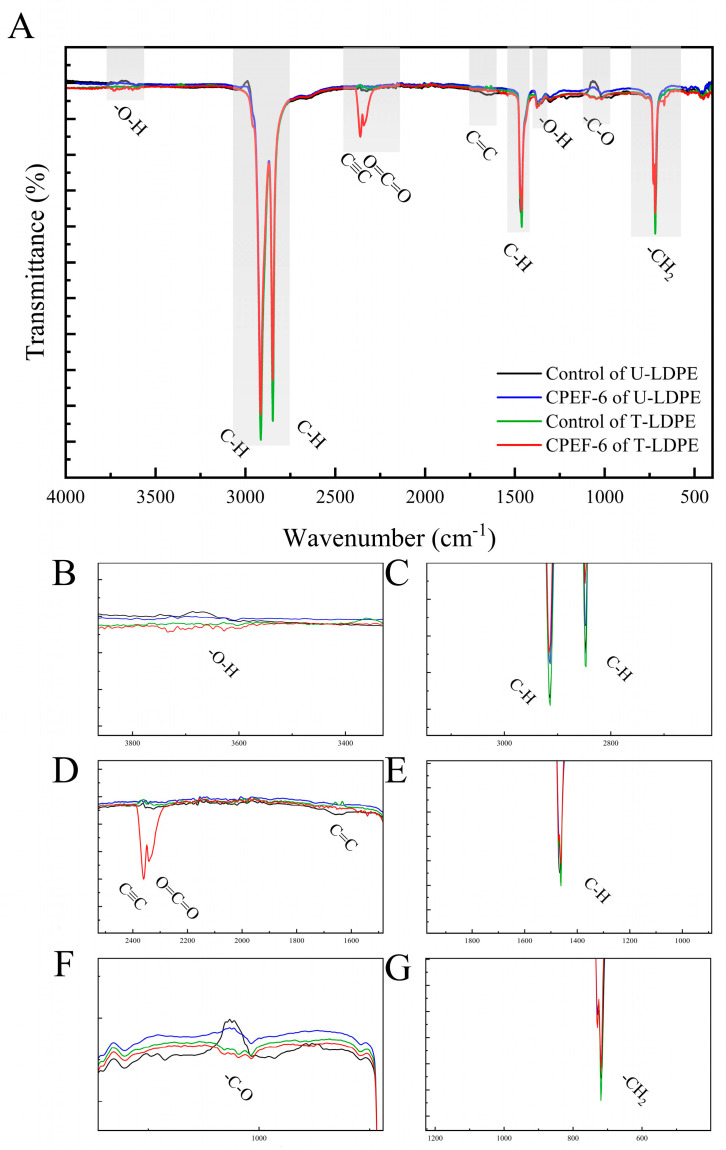
(**A**) FTIR spectrum of U-LDPE and T-LDPE biodegradation after 30 days of incubation. (**B**–**G**) Partial regions in the FTIR spectrum.

The surface structure changes of the U-LDPE and T-LDPE after inoculation with CPEF-6 were determined using FTIR spectroscopy with a frequency range from 400 to 4000 cm^−1^_,_ and the data was then processed using Origin2023. The FTIR spectra of the virgin and biodegraded LDPE sheets are shown in Figure 7.

For the control, critical characteristic absorption bands were assigned from 3752 to 3614 cm^−1^ (-O-H stretching) (Figure 7B), 2914 cm^−1^ (-CH asymmetric stretching) (Figure 7C), 2847 cm^−1^ (-CH symmetric stretching) (Figure 7C), 1462 cm^−1^ (-CH bending deformation) (Figure 7E), 1375 cm^−1^ (-O-H stretching), and 719 cm^−1^ (rocking vibrational mode of -CH_2_) (Figure 7G). For U-LDPE that was biodegraded by CPEF-6 for 30 days, the FTIR spectra of other peaks decreased, except from 3752 to 3614 cm^−1^ and at 1375 cm^−1^. For T-LDPE inoculated with CPEF-6, the spectral changes of each peak were the largest. In addition, the other three groups of data all displayed peak fluctuations from 1690 to 1615 cm^−1^ (C=C stretching) (Figure 7D) compared to the untreated U-LDPE. The peaks from 3752 to 3614 cm^−1^ and from 1140 to 994 cm^−1^ (C–O stretching) (Figure 7F) showed significant fluctuations in both groups of T-LDPE. In particular, in the T-LDPE after 30 days of biodegradation by CPEF-6, a new peak appeared from 2395 to 2281 cm^−1^ (C≡C stretching and O=C=O stretching) (Figure 7D), which is clearly distinct from other spectral data. 

### 3.7. Laccase Activity

**Figure 8:**
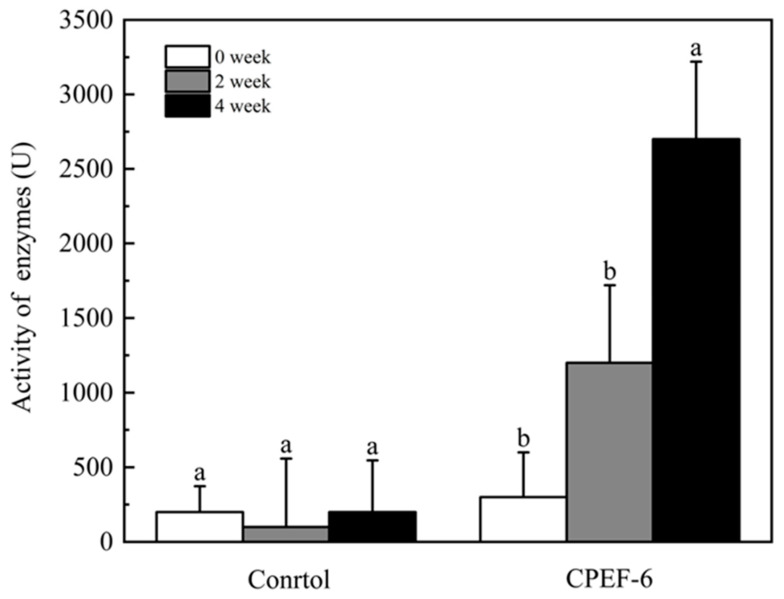
The enzyme activity of laccases in the culture supernatant. Different letters on the error bars represent significant change within sampling weeks (*p* < 0.05). Mean values with the same letter are not significantly different among treatments.

Microorganisms can secrete a variety of extracellular enzymes into the environment [42]. Laccase is one of the main fungal enzymes involved in polyethylene biodegradation [43]. The ABTS method was used to assay the activity of extracellular laccase in the supernatant of MSM-LDPE (T-LDPE) culture. During the co-culture growth of CPEF-6 with T-LDPE, we detected the production of laccase, and the activity of laccase gradually increased and reached 2700 ± 520 U/L in the fourth week (Figure 8).

## 4. Discussion

Biodegradation is the most effective method to control plastic pollution [13]. Studies have shown that PE degrading strains may be identified and those with potential PE degrading capacity can be screened out by observing turbidity in the medium [44]. Fungi release extracellular enzymes outward through the mycelia, converting macro and organic molecules into smaller organic compounds, which are then utilized as nutrients by microorganisms, and finally degraded into CO_2_ and H_2_O [14]. The degradation capability can also be demonstrated by examining the attachment of mycelia to the LDPE sheets. Here, we preliminarily verified the potential biodegradability of the fungal strain CPEF-6 on LDPE based on its growth and the amount of attachment to LDPE sheets (Figure 1). 

According to morphological observations and phylogenetic analysis (Figure 2 and Figure 3), the strain CPEF-6 is a member of the genus *Cladosporium* and shares the highest similarity with *Cladosporium basi-inflatum* CBS 822.84. *Cladosporium* sp., commonly found in soil, is a highly efficient fungal polymer-degrading fungus capable of growing on mediums with PU as the sole carbon source [45]. Although the ability of *Cladosporium* sp. to degrade LDPE has never been reported, some strains of *Cladosporium* sp. are thought to be capable of degrading complex polymers. Therefore, it is not surprising that strain *Cladosporium* sp. CPEF-6 had the ability to degrade the LDPE film. In a 2017 study, researchers isolated 10 strains of fungi from the waters of the Red Sea in an effort to identify fungi capable of degrading LDPE. *A. fumigatus* demonstrated an exceptional capacity for LDPE degradation, with a weight loss of 20.5% after 30 days of culture [46]. In order to compare and assess the LDPE degradability of strain *Cladosporium* sp. CPEF-6, we utilized *A. fumigatus* CPEF-7 as a reference strain. Our results show that both strains CPEF-6 and CPEF-7 have the ability to degrade LDPE sheets, while CPEF-6 (0.30 ± 0.06%) has a superior degradation ability than CPEF-7 (0.20 ± 0.01%) (Figure 4A). The properties of the polymer itself affect its degradation rates, such as molecular weight, crystallization rate, melting temperature, and additives added to the polymer [47]. The low weight loss may be due to the properties of the LDPE sheets used, which affects the degradation rate. In our experiments, after 30 days of CPEF-6 incubation, the percent of weight loss of T- LDPE sheets exceeded 30% compared to those of U-LDPE sheets (Figure 4). These findings suggest that T-LDPE is more susceptible to degradation by CPEF-6, and this result is consistent with those of other studies [48]. As a result of the heat treatment, the long chain backbones of the LDPE begin to separate and interact with each other, changing the properties of the complex polymer [49]. 

Fungi have complex metabolic activities during growth, and these metabolisms are directly associated with the biodegradation of LDPE. Throughout the incubation period, the biodegradation of LDPE to other components is mainly caused by fungal enzymes-induced biochemical reactions, which result in pH changes in the culture medium [39]. In both data sets where CPEF-6 was involved in the degradation of U-LDPE and T-LDPE, the pH decreased (Figure 5) significantly (*p* < 0.05). This was also confirmed by Ojha et al. [18]. The result may be that the strain produces small amounts of organic acids during the degradation of LDPE, affecting the pH of the culture broth and making it weakly acidic. The pH of biodegraded T-LDPE continued to decrease, dropping to 6.15 ± 0.02 in the fourth week (Figure 5B), and the decrease was slightly more significant than that of U-LDPE, confirming that LDPE sheets are more susceptible to microbial decomposition after heat treatment. Many studies have also shown that fungi are more capable of degrading polymers under acidic conditions [50], while in many studies of the fungal degradation of polyethylene, the pH has also been found to reduce to more acidic levels. For example, the fungus *Rhizopus oryzae* NS5 had an initial pH of 5.1 ± 0.27 during the degradation of polyethylene, but the pH dropped to 4.2 ± 0.30 after 30 days of incubation [50]. Consequently, the pH changes associated with PE functional groups and fungal enzymes serve as a significant reference point for the study of LDPE degradation.

The LDPE degradation was further authenticated using ESEM and FTIR. ESEM allows the observation of the superficial growth of fungal mycelium and the biodegradation of polyethylene, as shown in Figure 6. The biodegradation of polyethylene can be clearly seen through the formation of pores on the polyethylene surface and the penetration and colonization of fungal mycelium on the surface, as illustrated in Figure 6. In most studies, plastic surface changes were assessed by visible changes in the “before” and “after” images of the SEM, which were mainly described as the formation of cracks, pores, and holes [34]. Khan et al. demonstrated the degradation of LDPE by *Penicillium citricola* and found cavities, wrinkles, and cracks on the surface of LDPE after 90 days of biodegradation by SEM [39]. Likewise, Kyaw et al. offered SEM pictures of LDPE after 90 days of biodegradation that showed the biodegradability of selected *Pseudomonas* strains [51].

The FTIR generally measures changes in functional groups on the surface of plastics after biodegradation [52]. The pure LDPE sheet shows characteristic absorbance peaks at 2914, 2847, 1462, and 719 cm^−1^, which correspond to CH_2_ bending and stretching vibrations of LDPE [53]. In the present study (Figure 7), the characteristic peaks at 2914 cm^−1^ and 2847 cm^−1^ indicate the asymmetric and symmetric C-H stretching vibration, respectively [54], while 1462 cm^−1^ and 719 cm^−1^ represent the -CH bending deformation, corresponding to the rocking vibrational mode of -CH_2_ [55]. The change in C-H indicates that the alkyl group is the backbone in polyethylene [56], and these four peaks of the FTIR spectra decreased for both U-LDPE and T-LDPE compared to the control group after 30 days of biodegradation by CPEF-6. Among them, the spectrum of T-LDPE showed the largest peak variation. Similar studies have reported a decrease in peak intensity from 2800 to 3000 cm^−1^ for microbially degraded polyethylene [57,58]. A broad peak appears from 3752 to 3614 cm^−1^, which is caused by -OH stretching [59], indicating hydroxyl group formation. This is because the acidic O-H functional group supports the depolymerization process of LDPE substrates under the action of microbial strains [50]. The changes observed only in two spectra of T-LDPE indicate that the change in -OH is related to the heat treatment. This is consistent with the variation in LDPE/LLDPE studied by Jaiswal et al. [60]. Most specifically, after the biodegradation of T-LDPE by CPEF-6, a distinct peak from 2395 to 2281 cm^−1^ appeared, indicating an increase in the weak stretching of the C≡C bond and an increase in the strong stretching of the O=C=O bond [61]. This suggests that T-LDPE is more easily degraded and utilized by CPEF-6 than U-LDPE, a result that is consistent with the higher weight loss, lower pH, and more pronounced polyethylene surface in the T-LDPE treatment group. In Khandare et al.’s study of LDPE biodegradation by marine bacteria, the peak was observed in all treatments, with the most pronounced increase in its intensity in the bacterial treatment [61]. In addition, some studies have also observed an increase in this peak in the same way as the increase in fatty acid formation [62]. This is consistent with our speculation that the decrease in pH value represents the production of organic acids. Except for the control group of U-LDPE, the other three data sets all showed peak fluctuations from 1690 to 1615 cm^−1^, representing the formation of more C=C [21], as shown in Figure 7. This indicates that both thermal treatment and biodegradation increased the double bonds of polyethylene in our experiments. The formation of double bonds due to oxidation and hydrolysis microbial enzymes is considered an intermediate step in biodegradation and biofragmentation [63]. The results are consistent with the changes reported by Mohy Eldin et al. after the degradation of polyethylene bags by *Aspergillus terreus* [21]. T-LDPE incubated with CPEF-6 had the presence of alkoxy and acyl C-O stretching bands, demonstrating the production of alcohols, ethers, acids, and esters. The hydroxylation inferred using the peak from 1140 to 994 cm^−1^ related to alkoxy group formation (C-O), and provides evidence for polyethylene hydrolysis [64]. Peixoto et al. also showed C-O stretching in the infrared spectra of PE film sheets after 90 days of bacterial incubation [65]. The FTIR data expose the different strategies employed by CPEF-6 to degrade U-LDPE and T-LDPE, as evidenced by the difference in the wave number and intensity of the absorption peaks (Figure 7). This can also be demonstrated in conjunction with variations in the rate of weight loss, pH, and changes in polyethylene surface. In general, CPEF-6 has the highest degradation capacity for T-LDPE according to our findings.

Fungi produce a range of non-specific enzymes that catalyze the degradation of various pollutants [6]. Laccases are a class of enzymes belonging to the family of blue copper oxidases [25]. They were first discovered in fungi by Bertrand and Laborde in 1896 [66,67]. Laccase oxidizes LDPE hydrocarbon backbones; gel permeation chromatography demonstrated that cell-free laccase incubation decreased the average molecular number and weight of LDPE by 15% and 20%, respectively [68]. *Trichoderma harzianum*, when involved in PE biodegradation, could produce both laccase and peroxidase [69]. After 28 days, *Aspergillus flavus* PEDX3 was able to depolymerize HDPE long chains; PEDX3’s ability to generate laccases and laccase-like multicopper oxidases can be attributed to this observation [44]. Thus, the results of laccase production by CPEF-6 in the degradation of T-LDPE (Figure 8) suggest that CPEF-6 biodegrades polyethylene by producing laccase. The laccase reacts with polyethylene to produce organic acids that lower the pH, which explains our conclusion mentioned above regarding pH change as well as the ESEM and FTIR changes. During polymer degradation, complex polymers are first broken down by enzymes into short chains or monomers, which are subsequently utilized by microorganisms [70].

## 5. Conclusions

Based on weight reduction, ESEM, and FTIR analysis, *Cladosporium basi-inflatum* CPEF-6 is the most efficient and elite LDPE deteriorating fungus of the studied strains. In U-LDEP, CPEF-6 caused a 0.30 ± 0.06% weight loss, which was greater than the 0.20 ± 0.01% weight loss caused by the reference group, *Aspergillus fumigatus* CPEF-7 (Figure 4). The 0.43 ± 0.01% weight loss of CPEF-6 following a pair heat treatment demonstrates that the pretreatment enhances the degradability (Figure 4). ESEM analysis of the surface of the degraded polythene revealed phenomena including cracks and holes, as well as the attachment of mycelium, indicating corrosion (Figure 6). FTIR analysis revealed that the C-H peaks at 2914 cm^−1^, 2847 cm^−1^, 1462 cm^−1^, and 719 cm^−1^ were diminished after fungal treatment. C=C (1690 to 1615 cm^−1^) was changed in all except the control group of U-LDPE (Figure 7). There were changes in C≡C and O=C=O (2395 to 2281 cm^−1^), alkoxy C-O (1140 to 994 cm^−1^), and hydroxyl-OH (3752 to 3614 cm^−1^) after heat treatment. Changes in these peaks are attributable to the consumption of the C-H chains of polyethylene by fungi, resulting in the producing of fatty acids, alcohols, ethers, etc., which indicates the depolymerization of LDPE. These findings demonstrate the bio-oxidation of CPEF-6, which led to the exploration of degradation enzymes, and CPEF-6 was also shown to produce laccase during the degradation of T-LDPE (Figure 8). Thus, this work suggests the development of a new fungal strain typically found in the soil for the degradation of LDPE and the reduction of the difficulty of polyethylene degradation through thermal treatment in order to mitigate the current threat of an overabundance of polymeric materials causing massive environmental pollution.

## Data Availability

Not applicable.

## Supplementary Materials

The following supporting information can be downloaded at https://www.mdpi.com/article/10.3390/jof9060605/s1. Figure S1: Morphological features of strain CPEF-7. (A) The colony morphology of strain CPEF-7 after 5 days of incubation on the obverse PDA plate. (B) The colony morphology of strain CPEF-7 after 5 days of incubation on the reverse PDA plate. (C-D) Conidiophore of strain CPEF-7 (from A and B, respectively); Figure S2: Neighbor-Joining tree of the fungal genus *Aspergillus* based on ITS sequences. Type strains are in bold. The tree was rooted to *Aspergillus clavatus* (CBS 513.65). The evolutionary history was inferred using the Neighbor-Joining method [37]. The phylogenetic tree was constructed using the MEGA 7 program. The supported values from the 1000 bootstrap copies are illustrated under their respective branches; Table S1: Growth of the strains and the attachment of strains on the LDPE sheet among the part of the strains (MSM-LDPE medium); Table S2: Strains used in the molecular phylogenetic analysis of CPEF-6 in this study; Table S3. Strains used in the molecular phylogenetic analysis of CPEF-7 in this study.
