# Supplementary material for: Biodegradation of Low Density Polyethylene by the Fungus Cladosporium sp. Recovered from a Landfill Site
Source: J Fungi (Basel). 2023 May 24;9(6):605. doi: 10.3390/jof9060605 (PMC10301175; doi:10.3390/jof9060605)
Supplement: Supplementary file 1 [file jof-09-00605-s001.zip › jof-2388471-supplementary.pdf]

Journal of *Fungi*

Article

Biodegradation of Low Density Polyethylene by the Fungus *Cladosporium* sp. Recovered from a Landfill Site

Zhu Gong [1,†], Long Jin [1,†], Xing-Ye Yu [1], Bao-Teng Wang [1], Shuang Hu [1], Hong-Hua Ruan [1], Yun-Ju Sung [2], Hyung-Gwan Lee [3,*] and Feng-Jie Jin [1,*]

[1] Co-Innovation Center for Sustainable Forestry in Southern China, College of Biology and the Environment, Nanjing Forestry University, 159 Longpan Road, Nanjing 210037, China; gongzhu01@njfu.edu.cn (Z.G.); isacckim@alumni.kaist.ac.kr (L.J.);

[2] Technology Transfer Centre, Korea Research Institute of Bioscience & Biotechnology (KRIBB), Daejeon 34141, Republic of Korea

[3] Cell Factory Research Centre, Korea Research Institute of Bioscience & Biotechnology (KRIBB), Daejeon 34141, Republic of Korea

* Correspondence: trustin@kribb.re.kr (H.-G.L.); jinfj@njfu.edu.cn (F.-J.J.); Tel.: +82-42-860-4318 (H.-G.L.); +86-25-8542-7210 (F.-J.J.)

† These authors contributed equally to this work.

Abstract: Low density polyethylene (LDPE) has been widely used commercially for decades; however, as a non-degradable material, its continuous accumulation has contributed to serious environmental issues. A fungal strain, *Cladosporium* sp. CPEF-6 exhibiting a significant growth advantage on MSM-LDPE (minimal salt medium), was isolated and selected for biodegradation analysis. LDPE biodegradation was analyzed by weight loss percent, change in pH during fungal growth, environmental scanning electron microscopy (ESEM), and Fourier transformed infrared spectroscopy (FTIR). Inoculation with the strain *Cladosporium* sp. CPEF-6 resulted in a 0.30 ± 0.06% decrease in the weight of untreated LDPE (U-LDPE). After heat treatment (T-LDPE), the weight loss of LDPE increased significantly and reached 0.43 ± 0.01% after 30 days of culture. The pH of the medium was measured during LDPE degradation to assess the environmental changes caused by enzymes and organic acids secreted by the fungus. The fungal degradation of LDPE sheets was characterized by ESEM analysis of topographical alterations, such as cracks, pits, voids, and roughness. FTIR analysis of U-LDPE and T-LDPE revealed the appearance of novel functional groups associated with hydrocarbon biodegradation as well as changes in the polymer carbon chain, confirming the depolymerization of LDPE. This is the first report demonstrating the capacity of *Cladosporium* sp. to degrade LDPE, with the expectation that this finding can be used to ameliorate the negative impact of plastics on the environment.

Keywords: biodegradation; *Cladosporium* sp. CPEF-6; LDPE degradation; laccase; plastic-degrading fungi

Figure S1. Morphological features of strain CPEF-7. (A) The colony morphology of strain CPEF-7 after 5 days of incubation on the obverse PDA plate. (B) The colony morphology of strain CPEF-7 after 5 days of incubation on the reverse PDA plate. (C-D) Conidiophore of strain CPEF-7 (from A and B, respectively).

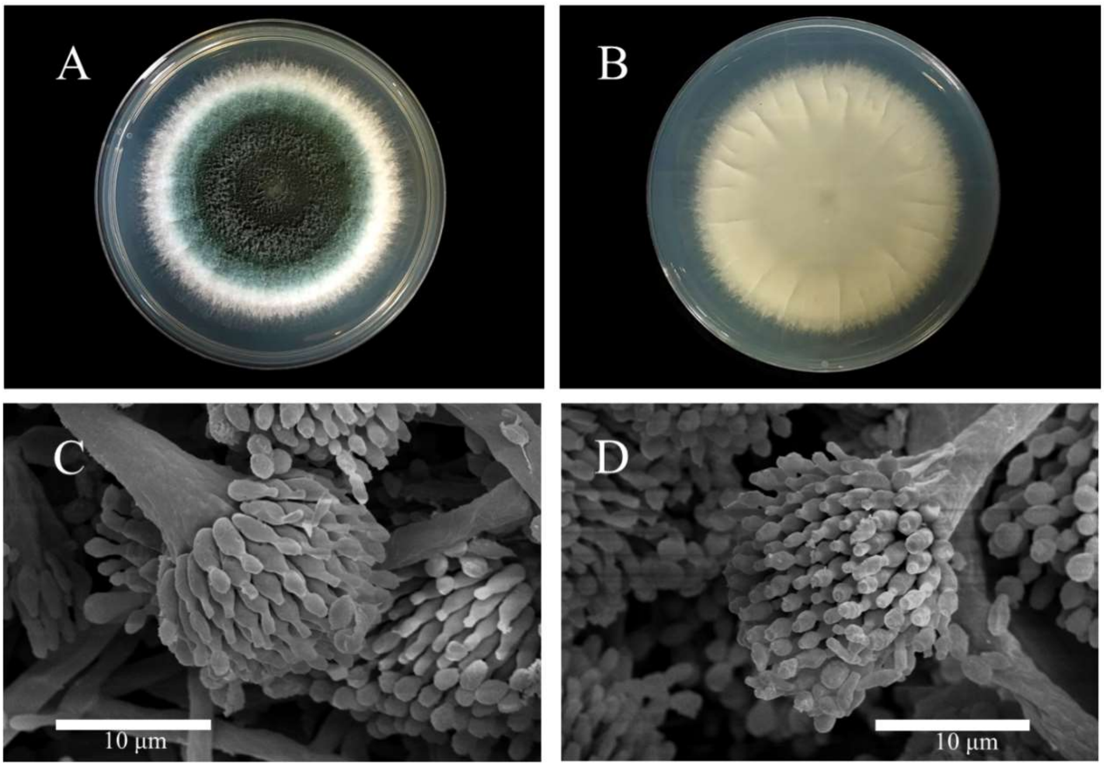

Figure S2. Neighbor-Joining tree of the fungal genus *Aspergillus* based on ITS sequences. Type strains are in bold. The tree was rooted to *Aspergillus clavatus* (CBS 513.65). The evolutionary history was inferred using the Neighbor-Joining method [37]. The phylogenetic tree was constructed using the MEGA 7 program. The supported values from the 1000 bootstrap copies are illustrated under their respective branches.

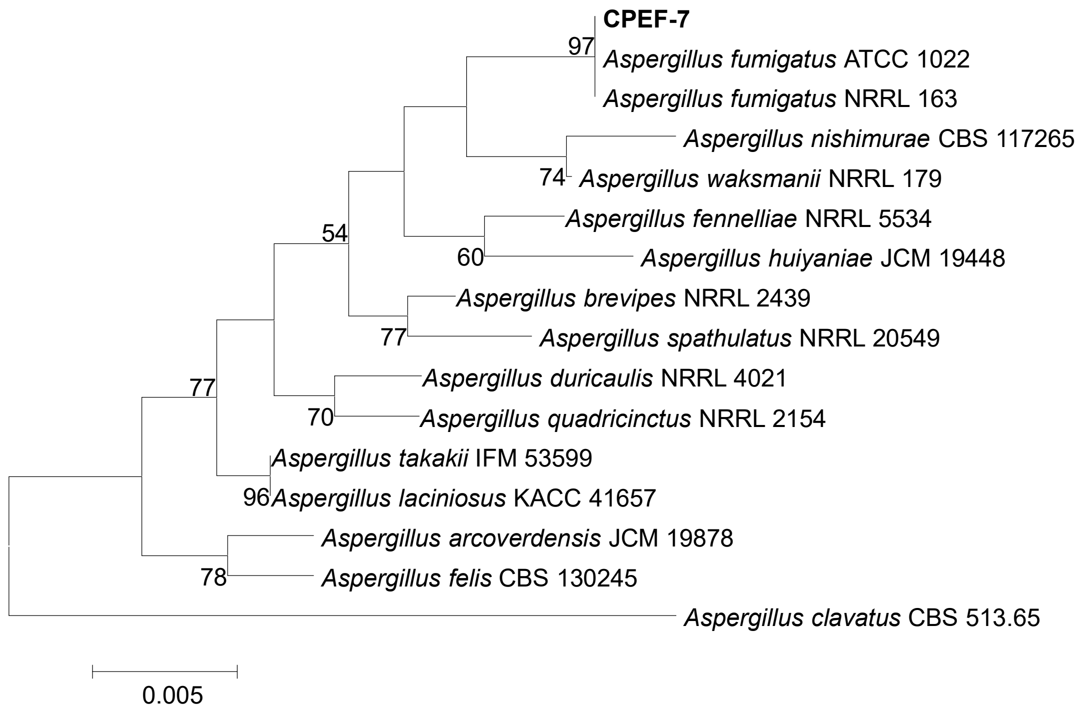

Table S1. Growth of the strains and the attachment of strains on the LDPE sheet among the part of the strains (MSM-LDPE medium).

Strain	Growth of the strains	The attachment of strains on the LDPE sheet
CK	-	-
CPEF-1	+	-
CPEF-3	+++	+
CPEF-6	+++	++
CPEF-7	+++	-
CPEF-8	+	-
PE2F-5	-	-
PE2F-6	+	-
PE4F-2	++	-
PE4F-3	++	+

'-' represents clear cultures, with no attachment to the LDPE sheet; '+' to '+++' represent slightly turbid to extremely turbid cultures and almost no strains to a large number of strains attached to the LDPE sheet.

Table S2. Strains used in the molecular phylogenetic analysis of CPEF-6 in this study.

Species	Collection' isolate number	GenBank accession number
		ITS
CPEF-6	………..	OQ651281
Cladosporium basi-inflatum^{T}	CBS 822.84	NR_111531.1
Cladosporium exasperatum^{T}	CPC 14638	NR_119843.1
Cladosporium langeronii^{T}	CBS 189.54	NR_111223.1
Cladosporium aphidis^{T}	CPC 13204	NR_120010.1
Cladosporium sphaerospermum^{T}	CBS 193.54	NR_111222.1
Cladosporium cycadicola^{T}	CBS 137970	NR_156279.1
Cladosporium dominicanum^{T}	CBS:119415	NR_119603.1
Cladosporium halotolerans^{T}	CBS:119416	NR_119605.1
Cladosporium endophyticum^{T}	MFLUCC 17-0599	NR_158360.1
Cercospora beticola^{T}	CBS 116456	NR_121315.1

Table S3. Strains used in the molecular phylogenetic analysis of CPEF-7 in this study

Species	Collection' isolate number	GenBank accession number
		ITS
CPEF-7	-	OQ651969
Aspergillus fumigatus[T]	ATCC 1022	NR_121481.1
Aspergillus fumigatus[T]	NRRL 163	EF669931.1
Aspergillus nishimurae[T]	CBS 117265	NR_137515.1
Aspergillus waksmanii[T]	NRRL 179	NR_135404.1
Aspergillus fennelliae[T]	NRRL 5534	NR_137490.1
Aspergillus huiyaniae[T]	JCM 19448	NR_151815.1
Aspergillus brevipes[T]	NRRL 2439	NR_135406.1
Aspergillus spathulatus[T]	NRRL 20549	NR_137480.1
Aspergillus duricaulis[T]	NRRL 4021	NR_135408.1
Aspergillus quadricinctus[T]	NRRL 2154	NR_137482.1
Aspergillus takakii[T]	IFM_53599	MN431378.1
Aspergillus laciniosus[T]	KACC 41657	NR_137443.1
Aspergillus arcoverdensis[T]	JCM 19878	NR_151816.1
Aspergillus felis[T]	CBS 130245	NR_138341.1
Aspergillus clavatus[T]	CBS 513.65	MH858693.1
